# Glycogen Synthase Kinase-3 regulates multiple myeloma cell growth and bortezomib-induced cell death

**DOI:** 10.1186/1471-2407-10-526

**Published:** 2010-10-04

**Authors:** Francesco Piazza, Sabrina Manni, Laura Quotti Tubi, Barbara Montini, Laura Pavan, Anna Colpo, Marianna Gnoato, Anna Cabrelle, Fausto Adami, Renato Zambello, Livio Trentin, Carmela Gurrieri, Gianpietro Semenzato

**Affiliations:** 1 Department of Clinical and Experimental Medicine, Hematology and Clinical Immunology Branch, University of Padua School of Medicine, Via Giustiniani 2 -35128-Padua, Italy; 2 Venetian Institute of Molecular Medicine, Centro di Eccellenza per la Ricerca Biomedica, Fondazione per la Ricerca Biomedica Avanzata, Via Orus 2 - 35129 - Padua, Italy

## Abstract

**Background:**

Glycogen Synthase Kinase-3 (GSK-3) α and β are two serine-threonine kinases controlling insulin, Wnt/β-catenin, NF-κB signaling and other cancer-associated transduction pathways. Recent evidence suggests that GSK-3 could function as growth-promoting kinases, especially in malignant cells. In this study, we have investigated GSK-3α and GSK-3β function in multiple myeloma (MM).

**Methods:**

GSK-3 α and β expression and cellular localization were investigated by Western blot (WB) and immunofluorescence analysis in a panel of MM cell lines and in freshly isolated plasma cells from patients. MM cell growth, viability and sensitivity to bortezomib was assessed upon treatment with GSK-3 specific inhibitors or transfection with siRNAs against GSK-3 α and β isoforms. Survival signaling pathways were studied with WB analysis.

**Results:**

GSK-3α and GSK-3β were differently expressed and phosphorylated in MM cells. Inhibition of GSK-3 with the ATP-competitive, small chemical compounds SB216763 and SB415286 caused MM cell growth arrest and apoptosis through the activation of the intrinsic pathway. Importantly, the two inhibitors augmented the bortezomib-induced MM cell cytotoxicity. RNA interference experiments showed that the two GSK-3 isoforms have distinct roles: GSK-3β knock down decreased MM cell viability, while GSK-3α knock down was associated with a higher rate of bortezomib-induced cytotoxicity. GSK-3 inhibition caused accumulation of β-catenin and nuclear phospho-ERK1, 2. Moreover, GSK-3 inhibition and GSK-3α knockdown enhanced bortezomib-induced AKT and MCL-1 protein degradation. Interestingly, bortezomib caused a reduction of GSK-3 serine phosphorylation and its nuclear accumulation with a mechanism that resulted partly dependent on GSK-3 itself.

**Conclusions:**

These data suggest that in MM cells GSK-3α and β i) play distinct roles in cell survival and ii) modulate the sensitivity to proteasome inhibitors.

## Background

GSK-3 is a pleiotropic serine-threonine kinase discovered for its involvement in insulin signaling. Two major isozymes (GSK-3α and GSK-3β) are known and conserved throughout the species [1]. This kinase is involved in cell proliferation and survival by controlling the Wnt/β-catenin and growth factors (GFs)-dependent pathways [2]. Constitutive GSK-3-mediated phosphorylation directs β-catenin to proteasome-mediated degradation [3]. Upon activation of Wnt signalling, GSK-3 activity is hampered and unphosphorylated β-catenin accumulates in the cytosol, translocates to the nucleus and promotes gene transcription and cell growth by acting as a co-activator of the transcription factors TCF/LEF [3,4]. GSK-3 is also inhibited by the action of the Phosphatidylinositol 3-OH kinase (PI3K)/AKT cell-survival pathway through phosphorylation on serine 21 (GSK-3α) and serine 9 (GSK-3β) [2]. Since Wnt/β-catenin and PI3K/AKT-dependent signaling pathways promote cell growth, GSK-3 has been considered a growth-suppressor. By contrast, GSK-3β has been found essential for cell survival by critically regulating NF-κB transcription factor activity and by protecting cells from TNFα [5] and TRAIL-induced apoptosis [6-9]. Moreover, while a number of studies demonstrated that GSK-3 may favor intrinsic apoptosis [10-12], other work showed that its inhibition could result in cancer cell apoptosis and growth arrest, in some cases due to an impaired NF-κB activity [8,13-15]. Taken together, several lines of evidence indicate that GSK-3 could play a twofold role in cell survival, depending on the different contexts (for instance, malignant versus non-malignant cells) or on whether apoptosis is started by intrinsic or extrinsic mechanisms [16]. However, a caveat for many of these studies is that GSK-3α and β isoforms were not evaluated separately.

Multiple myeloma (MM) is an incurable malignancy of plasma cells (PC) that clonally expand in the bone marrow (BM) [17]. Signaling pathways that might lead to GSK-3 inactivation and to NF-κB activation have been implicated in MM pathogenesis [18,19]. For instance, interleukin-6 (IL-6) production by BM stromal cells (BMSC), which stimulates malignant PC growth and expression of adhesion molecules, is NF-κB-dependent [20]. Insulin-like Growth Factor-I (IGF-I), an important growth and chemotactic factor for MM cells [21,22], can activate both PI3K/AKT and NF-κB. Also, Tumor Necrosis Factor-α (TNFα) produced in the tumor microenvironment can lend MM cells the ability to escape apoptosis by up regulating NF-κB dependent anti-apoptotic molecules [23]. Whether GSK-3 plays a role in NF-κB activation in MM and other blood tumors upon these and other stimuli is largely unknown. Moreover, the Wnt/β-catenin pathway causes MM cells proliferation [24], suggesting that secreted Wnt proteins in the BM microenvironment may act as GFs for malignant plasma cells. Furthermore, active Wnt signaling is also crucially involved in osteoblast differentiation [25,26]. Interestingly, the Wnt antagonist DKK1 is over expressed in MM patients displaying impaired bone formation and bone lytic lesions [27]. In this scenario, GSK-3, by inhibiting Wnt signaling, should be a growth brake for MM cells but also a negative regulator of osteoblastogenesis. Thus, the use of GSK-3 inhibitors to by-pass the DKK1-mediated Wnt signaling block on osteoblast precursors - that has been proposed with the aim to slow down the progression of myeloma bone disease [28,29] - could be hazardous. It is hence important to study both the role of GSK-3 in MM cell growth as well as the effects of its inhibition on the MM cell-tumor microenvironment interactions. Likewise, the investigation of the potential regulation of drug-induced cytotoxicity by GSK-3 - expected to occur in light of its role in the NF-κB signaling - would provide hints to determine the feasibility of this potentially useful therapeutic approach in MM therapy.

With this as a background, the aim of our study was to analyze GSK-3 role in MM cell growth and survival. By using selective ATP-competitive small chemical GSK-3 inhibitors as well as gene knock down by RNA interference, we investigated the consequences of GSK-3 (α and β) down regulation in MM cells. We found that treatment of MM cells with GSK-3 inhibitors and GSK-3β knock down caused growth arrest and apoptosis by perturbing pivotal signaling pathways. We also found that the GSK-3 inhibitors and GSK-3α knock down enhanced the anti-MM cytotoxic effect of bortezomib, a clinically used proteasome inhibitor.

## Results

### GSK-3 expression in MM cells

**Figure 1:**
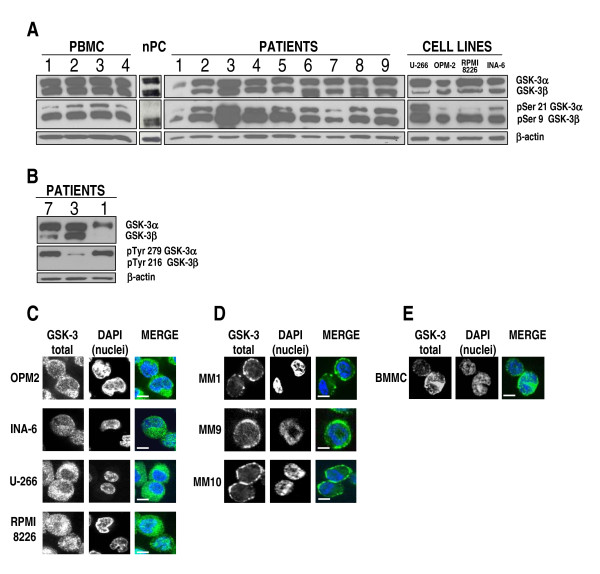
**GSK-3 expression, phosphorylation and localization in MM cells**. (A) WB for total GSK-3α and β (top panels), phosphorylated GSK-3α (on Ser 21) and GSK-3β (on Ser 9) (middle panels), and β-actin as loading control (bottom panels) in (from left to right): 4 PBMC samples of healthy subjects, non-malignant *in vitro*-generated PC (nPC), CD138^+ ^purified PC from 9 MM patients, 4 MMCLs; (B) WB for total GSK-3α and β (top panels), phosphorylated GSK-3α (on Tyr 279) and GSK-3β (on Tyr 216) (middle panels) and β-actin as loading control (bottom panels), in three primary MM plasma cell samples (MM 7, 3, 1). (C-E): GSK-3 immunostaining and confocal microscopy analysis in MMCLs OPM-2, INA-6, U-266 and RPMI-8226 (C), three CD138+ primary malignant PC from patients (MM1, MM9, MM10) (D) and BMMC (E). In the merged images GSK-3 is detected by green fluorescence and nuclei are in blue. Scale bars = 10 μm. For all the images: 600× magnification, oil objective.

GSK-3α/β (hereafter referred as GSK-3) activity is mainly inhibited through AKT [30] or S6K1 kinase-dependent [31] phosphorylation on Ser 9 (GSK-3β) and 21 (GSK-3α). Since MM cells receive signals that activate these two kinases, we investigated the expression of total and serine-phosphorylated (inactive) GSK-3 in these cells. Protein lysates were obtained from PBMC of 4 healthy subjects, normal *in vitro *generated PC (nPC), 9 primary MM samples (CD138^+ ^malignant PC), 4 MM cell lines (MMCLs); immunoblot analysis of total and serine-phosphorylated GSK-3 was then performed. As shown in Fig. 1A, we noticed an abundant expression of total GSK-3 in PBMC and nPC, both of the α and of the β isoform. In some MM cells from patients (namely cases 1, 2, 7 and 9) the GSK-3β isoform was moderately less expressed. This difference was seemingly more evident in the U-266, RPMI- 8226 and INA-6 cell lines.

### GSK-3 phosphorylation in MM cells

We observed that the GSK-3β isoform resulted to be more phosphorylated on Ser 9 than GSK-3α on Ser 21, both in normal and in malignant cells, suggesting that its catalytic activity is buffered down. Since GSK-3 tyrosine phosphorylation indicates its activation, to further confirm these results, we performed WB analysis of the levels of phopsho-Tyr 279 GSK-3α and phospho-Tyr 216 GSK-3β on protein lysates of patients 7, 3 and 1. As shown in Fig. 1B, primary MM cells from three patients displayed a prevailing tyrosine phosphrylation of GSK-3α, whereas the levels of phospho-Tyr GSK-3β were barely detectable.

### GSK-3 subcellular localization in MM cells

We next analyzed the intracellular localization of GSK-3 in malignant PC by confocal immunofluorescence (IF). In resting non-malignant cells GSK-3 localizes mostly in the cytoplasm and smaller pools of the kinase are present in the nucleus and other organelles where they exert specialized functions [32,33]. However, in some tumors, GSK-3β was found localized mostly in the nuclear compartment [14,34]. In MMCLs (OPM-2, INA-6, U-266 and RPMI-8226) GSK-3 was present in the cytosol (more in OPM-2 and U-266) and in the nucleus (more in INA-6 and RPMI-8226 cells); in primary malignant PC from 3 patients (indicated as MM1, MM9 and MM10) GSK-3 was found to be scarcely localized in the nucleus, much more in the cytosol and also close to the cell membrane (Fig. 1C and 1D); in control BMMC the intracellular distribution of GSK-3 was mostly cytosolic and/or with a speckled-like nuclear pattern and with very little presence at the plasma membrane edge (Fig. 1E).

### Effects of GSK-3 inhibitors on GSK-3 activity in MM cells

**Figure 2:**
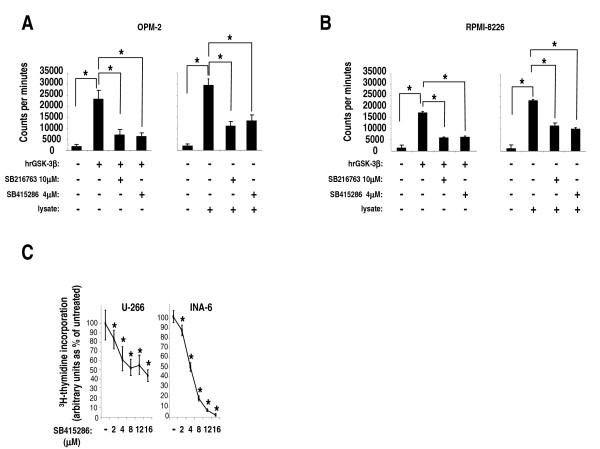
**Effects of GSK-3 inhibitors on MM cell proliferation**. (A, B) Graphs showing representative *in vitro *kinase assays performed using human recombinant GSK-3β (hrGSK-3β) and total protein lysates (+ = 1 μg, ++ = 4 μg) of OPM-2 cells (A) and RPMI-8226 cells (B) that were cultured with the two specific GSK-3 inhibitors, SB216763 at 10 μM and SB415286 at 4 μM. The graphs show a remarkable inhibition of both hrGSK-3β protein (leftmost graphs) and endogenous GSK-3 kinase activity (rightmost graphs) (y axis: counts per minute). Data represent mean ± SD, *n *= 3. * indicates *p *< 0.05 by one-way ANOVA test. (C) Dose-response graphs of U-266 (left) and INA-6 cells (right) incubated for 48 hours with increasing concentrations of SB415286. Cell proliferation was assessed by ^3^H-thymidine-incorporation assay. Data represent mean ± SD, *n *= 4, * indicates *p *< 0.05 by one-way ANOVA test.

To investigate GSK-3 function in MM cells we used the well characterized *in vitro *and *in vivo*, ATP-competitive, GSK-3 chemical inhibitors SB216763 and SB415286. These compounds were tested in previous studies for their activity against a panel of several different kinases (> 20), displaying a fairly high specificity for GSK-3α and GSK-3β [35,36]. First, we tested the efficacy to inhibit GSK-3 by culturing MM cells OPM-2 and RPMI-8226 in the presence of the two compounds. These two cell lines were chosen since they displayed lower phospho-serine GSK-3α/β levels (and thus higher activity) than the others. Total GSK-3 activity (α plus β isozymes) was then determined on cellular protein lysates after 24 hours using a radioactive kinase assay. The inhibitory activity of SB216763 and SB415286 was assayed against human recombinant GSK-3β (hrGSK-3β) protein, as a control. As shown in Fig. 2A (OPM-2 cell line) and Fig. 2B (RPMI-8226 cell line), both the inhibitors hampered hrGSK-3β (leftmost graph) and endogenous (rightmost graph) GSK-3 activity (*p *< 0.05, n = 3). Similar results were obtained using U-266 and INA-6 cells (not shown), demonstrating that these compounds can efficiently inhibit the active pool of GSK-3 in MM cells.

### GSK-3 down regulation decreases MM cell growth and survival

We next checked the effects of the GSK-3 inhibitors on malignant PC growth by treating MM cells with increasing concentrations of SB415286 or SB216763. In these conditions, all the MMCLs tested displayed a dose-dependent decrease of their growth as determined by reduced thymidine incorporation (U-266, INA-6) (*p *< 0.05, n = 4) (Fig. 2C) and BrdU staining (RPMI-8226, U-266, INA-6) (*p *< 0.05, n = 5)(data not shown), indicating that GSK-3 may function as a growth-promoter in these cell lines. Data were represented as percent of untreated cells (made equal to 100% of incorporation of ^3^H-thymidine evaluated as counts per minutes or 100% of BrdU fluorescence staining evaluated by FACS analysis, as specified in Methods).

**Figure 3:**
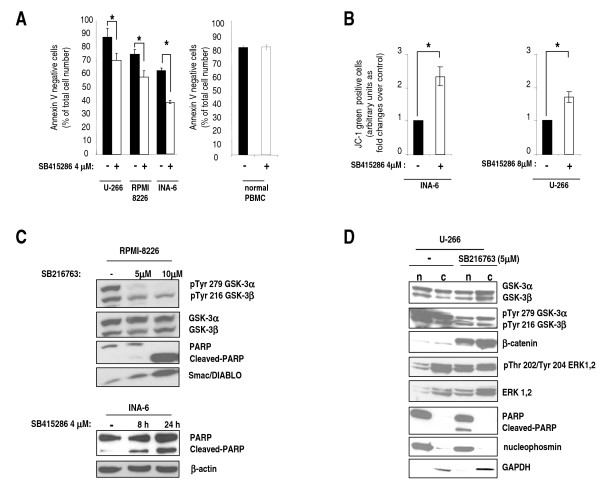
**GSK-3 inhibition causes MM cell apoptosis and disruption of the mitochondrial membrane potential**. (A) Histograms showing the percentage of surviving (annexin V negative) U-266, RPMI-8226 and INA-6 cells (left graph) and of normal PBMC control cells (right graph) after treatment without (black bars) or with (white bars) 4 μM SB415286 for 48 hours. Data represent mean ± SD, *n *= 5 (cell lines) or 4 (PBMC). * indicates *p *< 0.05 by Student's t-test. (B) Histograms showing FACS analysis of the amount of JC-1 monomer-containing, green fluorescent-INA-6 cells (left graph) or U-266 cells (right graph), treated with the same conditions as in A. Data are expressed as ratio over untreated cells. Data represent mean ± SEM, *n *= 5. * indicates *p *< 0.01 by Student's t-test. (C) Representative WB analysis of phospho Tyr 279 GSK-3α and phospho Tyr 216 GSK-3β, total GSK-3, PARP cleavage and Smac/DIABLO protein expression on cell lysates of RPMI-8226 cells grown with 5 μM or 10 μM SB216763 for 24 hours (top panels) and WB analysis of PARP protein in INA-6 cells grown with 4 μM SB415286 for 8 and 24 hours (bottom). β-Actin is used to check protein loading. (D) Representative WB analysis of nuclear (n) and cytosolic (c) proteins from U-266 cells untreated or treated with 5 μM SB216763, showing the levels of total GSK-3, Tyr phosphorylated GSK-3, β-catenin, total ERK 1/2, Thr/Tyr phosphorylated ERK 1/2, PARP and nucleophosmin and GAPDH as loading controls.

Then, we investigated whether apoptosis could be one mechanism of growth inhibition in GSK-3 inhibitor-treated MM cells. Annexin V staining and FACS analysis experiments using U-266, RPMI-8226 and INA-6 cells showed that treatment with SB415286 resulted in a moderate though significant reduction of cell viability (*p *< 0.05, n = 5) (Fig. 3A, graph on the left). Importantly, at this concentration of SB415286 normal PBMCs did not display apoptosis as compared to untreated samples (Fig. 3A, graph on the right). To further validate the above results, we measured the electrochemical gradient across the mitochondrial membrane in the absence or presence of GSK-3 inhibition (4 or 8 μM SB415286). Experiments carried out with the JC-1 dye [37] showed that GSK-3 inhibition in INA-6 and U-266 cells caused the disruption of the mitochondrial membrane potential (increase of the number of cells stained with JC-1 green-fluorescent monomers, *p *< 0.01, n = 5) (Fig. 3B). Furthermore, WB analysis of protein lysates obtained from SB216763-treated RPMI-8226 cells revealed that GSK-3 inhibition was associated with a significant reduction of the amount of phospho-Tyr 279 GSK-3α and phospho-Tyr 216 GSK-3β, indicating that the compound efficiently inhibited the autocatalytic activity of GSK-3, in agreement with previous studies [38,39] and with our above described *in vitro *kinase assays. To note, SB216763 caused activation of the intrinsic mitochondria-dependent apoptosis evidenced by Poly (ADP ribose) polymerase (PARP) cleavage and a significant increase of Smac/DIABLO cytoplasmic protein levels (Fig. 3C, top panels). Also SB415286-treatment of INA-6 cells caused apoptosis and PARP cleavage, time-dependently (Fig. 3C, bottom panels).

### Inhibition of GSK-3 increases β-catenin and phospho-ERK1, 2 levels in MM cells

Next, we investigated the effects of SB216763 and SB415286 (not shown) treatment of U-266 MM cells on GSK-3-related signaling pathways by WB experiments using cytoplasmic and nuclear protein lysates (Fig. 3D). The GSK-3 inhibitors caused a significant increase of β-catenin levels, in the cytoplasm and in the nucleus, as expected - again showing a remarkable inhibition of GSK-3 Tyr phosphorylation. Interestingly, in the presence of GSK-3 inhibition an accumulation of GSK-3 in the cytosol was observed. We also looked at the ERK1/2 pathway, an important proliferative cascade for MM cells, since GSK-3 was shown to negatively regulate it in the myocardial muscle [40] and in immune cells [41]. Indeed, ERK1/2 phosphorylation was also affected by GSK-3 inhibition - even if in a slighter fashion - with mild increase of the phospho-Thr202/Tyr204 ERK1, 2 nuclear pool, indicating a role - likely minor - for GSK-3 in this context (Fig. 3D).

### Molecular *sequelae *and effects of GSK-3 inhibition on bortezomib-dependent cytotoxicity in MM cells

**Figure 4:**
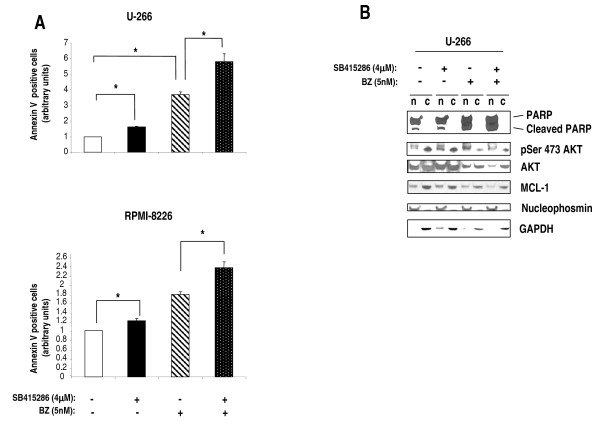
**GSK-3 inhibition increases MM cell sensitivity to bortezomib-induced apoptosis**. (A) Histograms summarizing the data on the percentage of apoptotic MM cells, evaluated by annexin V staining and FACS analysis, left untreated (white bars) treated with the GSK-3 inhibitor SB415286 (4 μM) (black bars), bortezomib (BZ) 5 nM (black and white dashed bars) or both (black, white-dotted bars). Top graphs: U-266 cells, bottom: RPMI-8226 cells. Data represent mean ± SEM, *n *= 3. * indicates *p *< 0.05 by one-way ANOVA test. (B) Representative WB analysis of nuclear (n) and cytosolic (c) proteins from U-266 cells untreated, treated with 4 μM SB415286, with 5 nM BZ or both, showing the levels of PARP and cleaved PARP, Ser 473-phosphorylated AKT, AKT and MCL-1. Nucleophosmin and GAPDH were used as nuclear and cytosolic loading controls, respectively.

Since GSK-3 controls MM cell survival, we next checked whether it could modulate the pro-apoptotic effects of the proteasome inhibitor bortezomib (BZ), a clinically used anti-MM agent that down modulates growth-promoting pathways, triggers mitochondria-dependent apoptosis [42] and interferes with MM cell-BMSC interactions [43]. To this aim, MM cells (U-266 and RPMI-8226) were treated with either 4 μM SB415286, either 5 nM BZ or both and annexin V staining and FACS analysis were used to quantify apoptosis. As shown in Fig. 4A, the simultaneous treatment of MM cells (U-266 and RPMI-8226) with the GSK-3 inhibitor and BZ caused a moderate but significant increase of apoptosis in both MM cell lines as compared with the apoptotic rate observed with single treatments (*p *< 0.05, n = 3 for both series of experiments). Immunoblot analysis of survival and BZ-regulated MM molecules confirmed that SB415286 caused an enhanced PARP cleavage in the nucleus compared to untreated samples. BZ exposure of MM cells was accompanied by a more pronounced PARP cleavage and, interestingly, by an increase of nuclear phospho-Ser 473 AKT levels, a reduction of total AKT and of the anti-apoptotic BCL-2 family member MCL-1 protein levels. These effects of BZ were already described in other studies [44,45], even though in certain cancer cell types BZ was shown to reduce phospho-Ser 473 AKT levels [46,47]. Remarkably, the treatment of MM cells with GSK-3 inhibitors together with BZ resulted in an even further increase of PARP cleavage and reduction of phospho-Ser 473 AKT levels, total AKT and MCL-1 protein levels (Fig. 4B).

### GSK-3α and β silencing by RNA interference unveils a different role for the two isoforms in MM cell survival

**Figure 5:**
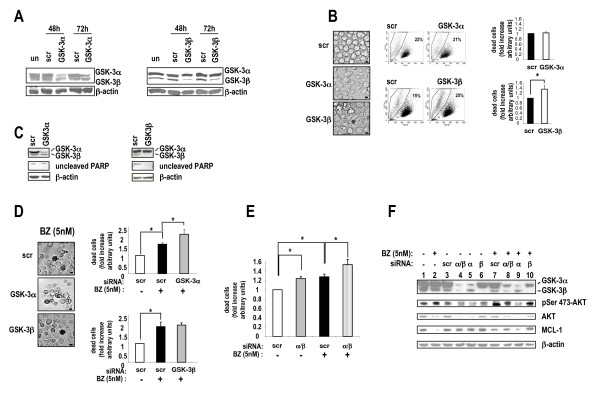
**Effects of GSK-3α and β knockdown by RNA interference on MM cell survival**. (A) WB analysis of the levels of GSK-3α (left panels) or GSK-3β (right panels) upon nucleofection with: un = untransfected; scr = scrambled siRNAs oligos; GSK-3α or GSK-3β=GSK-3α or GSK-3β-directed siRNAs oligos. (B, left panels) Light microscopy microphotographs of U-266 cells transfected with scrambled (scr), GSK-3α or GSK-3β-directed siRNAs oligos. Scale bars = 10 μm. (B, middle plots) Representative dot plot graphs and FACS analysis of U-266 cells transfected with scrambled (scr), GSK-3α or GSK-3β-directed siRNAs oligos. (B, right panels) Histograms summarizing the results of FACS analysis of U-266 cells transfected with scrambled (scr) siRNAs oligos, GSK-3α (top) or GSK-3β (bottom)-specific siRNAs oligos,. In y-axis dead cells are cells displaying a low FSC/high SSC cyto-morphological profile. Data represent mean ± SEM, *n *= 6. * indicates *p *< 0.05 (Student's t test). (C) Representative WB analysis of the levels of unprocessed PARP after nucleofection of U-266 cells with: scrambled, GSK-3α or GSK-3β siRNAs oligos. (D) Light microscopy microphotographs and histograms summarizing the results of FACS analysis of U-266 cells transfected with scrambled (scr), GSK-3α or GSK-3β-directed siRNAs oligos and treated with 5 nM BZ. In y-axis dead cells are cells displaying a low FSC/high SSC cyto-morphological profile. Data represent mean ± SEM, *n *= 3. * indicates *p *< 0.05 (one-way ANOVA test). (E) Histogram plot summarizing the results of the FACS analysis of U-266 cells transfected with scrambled (scr) or GSK-3α/GSK-3β-directed siRNA oligos and treated with 5 nM BZ. In y-axis dead cells are cells displaying a low FSC/high SSC cyto-morphological profile. Data represent mean ± SEM, *n *= 3. * indicates *p *< 0.05 (one-way ANOVA). (F) WB analysis of GSK-3α, GSK-3β, AKT, Ser 473-phosphorylated AKT and MCL-1 protein levels in U-266 cells untransfected (lane 1), treated with 5 nM BZ (lane2), transfected with scrambled (scr, lane 3) or GSK-3α/β (lane 4), GSK-3α (lane 5) or GSK-3β (lane 6)-directed siRNAs oligos without (-, lanes 3-6) or with (+, lanes 7-10) exposure to 5 nM BZ for 18 hours.

To further analyze the role of GSK-3 in MM cell growth we performed RNA interference experiments to knock down GSK-3α and GSK-3β protein expression levels in U-266 cells. As shown in Fig. 5A, we could achieve a significant reduction of GSK-3α (left panel) and GSK-3β (right panel) protein levels, peaking 48 hours and lasting as long as 72 hours after U-266 MM cells nucleofection with specific GSK-3α or GSK-3β-directed small interfering RNA oligos. The efficiency of transfection was high (transfected cells ranged from 80% to > 90%) in all the experiments performed (not shown). The control samples, either un-transfected or transfected with blank buffer or with scrambled RNA oligonucleotides did not show significant alterations of the protein kinase levels. Since as markers of nucleofection efficiency we used fluorescent RNA oligos that had an emission spectrum overlapping with those of propidium iodide, annexin V and JC-1, we used light microscopy as an alternative mean to analyze cell viability; this assay revealed that GSK-3β-silenced cells were more irregularly-shaped with picnotic nuclei as compared to cells transfected with scrambled or GSK-3α-targeting siRNAs (Fig. 5B, left panels). Moreover, the quantitative analysis of cellular morphology and granularity by FACS showed that GSK-3β-silenced samples displayed approximately a 1.4 fold increase in the percentage of dying [low forward scatter (FSC)/high side scatter (SSC)] cells as compared to samples transfected with scrambled siRNAs (*p *< 0.05, n = 6). This effect was not evident when GSK-3α was knocked down (Fig. 5B, middle panels; in the rightmost graphs data are summarized in histogram plots). In addition, silencing of GSK3α did not cause a substantial reduction of the unprocessed PARP protein as opposed to silencing of GSK-3β, suggesting that, at basal growing conditions, only GSK-3β may partially sustain MM cell survival (Fig. 5C).

### GSK-3α knock down empowers bortezomib-induced cell death

To confirm the results obtained with BZ and GSK-3 inhibitors, we carried out RNA interference experiments of GSK-3α and GSK-3β subunits in U-266 MM cells; 48 hours after transfection with the targeting siRNAs, cells were grown for additional 18 hours with or without BZ. Cells were then analyzed by light microscopy and FACS for morphological changes indicative of decreased viability, as above described. Treatment with BZ 5 nM caused marked apoptosis. Interestingly, knock down of GSK-3α, but not of GSK-3β, was associated with an increase of BZ-induced cell death, as evidenced by more irregularly-shaped cells with picnotic nuclei by light microscopy (Fig. 5D, left panels). Moreover, we did not observe significant changes in the percentage of low-FSC/high-SSC cells by FACS analysis of GSK-3β-silenced and BZ-treated cells (Fig. 5D, right panels, bottom graph), whereas we could detect a statistically significant decrease of viability in the GSK-3α-silenced and BZ-treated samples (*p *< 0.05, n = 3) as compared to scrambled oligos-transfected controls (Fig. 5D, right panels, top graph). We next analyzed U-266 cell viability upon silencing of both the GSK-3 isoforms and BZ treatment. As shown in the graph in Fig. 5E, RNA interference-mediated knock down of GSK-3α and GSK-3β expression was associated with decreased MM cell viability (*p *< 0.05, n = 3). Remarkably, silencing of GSK-3α and GSK-3β also augmented the cytotoxic effect of BZ (*p *< 0.05, n = 5), confirming the results observed with the chemical inhibitors.

### AKT and MCL-1 are targets of GSK-3 in MM cells

We next checked whether silencing of GSK-3 could lead to alterations of BZ-controlled pathways, as observed with the small chemical inhibitors. Immunoblot analysis showed a substantial reduction of GSK-3β or GSK-3α isoforms in these experiments (Fig. 5F, lanes 4-6, 8-10). BZ caused a reduction of AKT and MCL-1 protein levels (lane 2). Noteworthy, we observed that in both GSK-3α and GSK-3α/β double-silenced cells, AKT protein levels were also reduced. As a consequence, silencing of GSK-3α or GSK-3α plus GSK-3β was seemingly associated with a slight reduction of phospho-Ser 473 AKT (lanes 4 and 5). This effect was not as obvious in GSK-3β-only silenced cells (lane 6). BZ treatment of GSK-3α and GSK-3α/β double-silenced cells was associated with a marked reduction of AKT protein levels (lanes 8, 9). BZ also caused an increase of phospho-Ser 473 AKT (lane 2, 7). However, when both or the GSK-3α isoforms were knocked down, this effect was decreased (lanes 8, 9). Intriguingly, it appeared that silencing of GSK-3α somehow favored whereas silencing of GSK-3β protected against BZ-induced AKT and, even more, MCL-1 degradation (Fig. 5F, lanes 7-10).

### Bortezomib induces GSK-3 activation and nuclear translocation

**Figure 6:**
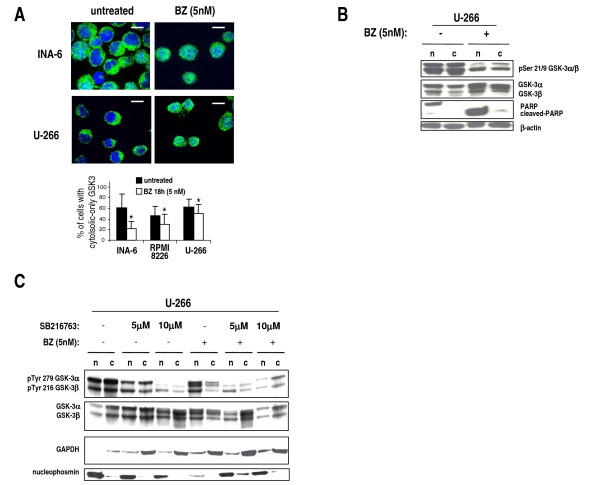
**Bortezomib modulates GSK-3 intracellular localization and activation**. (A) Immunofluorescence microscopy of INA-6 and U-266 cells untreated or treated with 5 nM BZ for 18 hours and stained for GSK-3 (green fluorescence) and the nuclei (DAPI, blue fluorescence) showing partial nuclear re-localization of GSK-3 upon BZ treatment. Scale bars = 10 μm. On the bottom, graph showing the percentage of MM cells scored as having cytosolic only GSK-3 in the untreated (black bars) or 5 nM BZ-treated (white bars) conditions. Data represent mean ± SD, *n *= 3. * indicates *p *< 0.05. (B) Representative WB analysis of phospho Ser 9 GSK-3β/Ser 21 GSK-3α and total GSK-3α/β in nuclear (n) and cytoplasmic (c) protein fractions from untreated (-) or 5 nM BZ-treated (18 hours) U-266 cells. β-Actin and nuclear PARP are used as markers for protein loading. (C) Representative WB analysis of phospho Tyr 279 GSK-3α/Tyr 216 GSK-3β and total GSK-3α/β in nuclear (n) and cytoplasmic (c) protein fractions from untreated (-) or 5 nM BZ-treated (+) (18 hours) U-266 cells, in the presence or absence of 5 μM or 10 μM SB216763. GAPDH and nucleophosmin were used as protein loading markers.

Since the above results suggest that GSK-3α can antagonize BZ-dependent cell death and as it has been demonstrated that GSK-3 serine and tyrosine phosphorylation levels and intracellular localization change upon different apoptotic *noxae *[48], we checked GSK-3 phosphorylation and cellular localization in MM cells upon treatment with BZ. While GSK-3 protein was detectable mostly in the cytosol of untreated cells, it was visible both in the nucleus and in the cytoplasm of cells treated with 5 nM BZ for 18 hours (Fig. 6A, a graph summarizing the scoring results is shown at the bottom; *p *< 0.05, *n *= 3 separate experiments). Most interestingly, WB experiments demonstrated a marked reduction of Ser 21/9 GSK-3 phosphorylation, both in the cytosol and in the nucleus, and a slight accumulation of total GSK-3 in the nuclear compartment, upon BZ treatment (Fig. 6B). In all the experiments, BZ was able to induce a significant amount of apoptosis, as assessed by WB analysis of PARP cleavage (Fig. 6B). Since GSK-3 nuclear translocation has been associated with Tyr phosphorylation, which in turn depends on GSK-3 activity itself, we next analyzed by WB the levels of phospho-Tyr GSK-3, total GSK-3 and nuclear/cytoplasm localization upon treatment with two concentrations (5 μM and 10 μM) of SB216763, 5 nM BZ or both. These experiments (Fig. 6C) revealed that SB216763 efficiently reduced the phospho-Tyr GSK-3 levels in the nucleus and in the cytoplasm; BZ caused a shift of phospho-Tyr GSK-3 and total GSK-3 in the nucleus and, interestingly, treatment with BZ plus SB216763, while it was still associated with a reduction of phospho-Tyr GSK-3, prevented the nuclear shuttling of the kinase.

## Discussion

The involvement of GSK-3 in cancer cell biology has recently been demonstrated. GSK-3 sustains the growth of malignant blood cells, like chronic lymphocytic leukemia (CLL) [34] and acute myeloid leukemia cells (AML) [49]. Our data demonstrated that GSK-3 regulates MM cell growth and survival. Importantly, we also found that GSK-3 modulates MM cell sensitivity to BZ-induced apoptosis.

The expression analysis revealed that, in most MM samples from patients and even more, in all the MMCLs, GSK-3β protein levels were lower than GSK-3α. This was not evident in normal controls (PBMC, nPC). GSK-3β also resulted more phosphorylated in Ser 9 than GSK-3α in Ser 21 both in normal and malignant samples; moreover, in the MM samples analyzed for GSK-3 Tyr phosphorylation, we found that GSK-3β was much less Tyr phosphorylated than GSK-3α (Fig. 1A). Since GSK-3β Ser 9-phosphorylation is associated with a reduction in the enzymatic activity whereas GSK-3β Tyr 216-phosphorylation indicates activation [30], these findings, even if to be validated on a larger number of primary MM samples, indicate that the GSK-3β isoform may be less active than GSK-3α in normal PBMC and nPC as well as in MM cells. In these latter cells, however, we observed also a reduction of GSK-3β expression that could contribute to a further down-regulation of its activity. Interestingly, GSK-3α and GSK-3β do not share the same physiological functions [50]. In particular, GSK-3α does not supply for the loss of GSK-3β-dependent NF-κB regulation in GSK-3β knock out cell, whereas it does in the regulation of the Wnt/β-catenin pathway [5]. Our expression studies also showed that in MM plasma cells from patients and some MMCLs (OPM-2, INA-6), GSK-3 was present in the cytosol and partly close to the cell surface, but rarely in the nucleus (Fig. 1B and 1C). Although in other types of cancer cells GSK-3 localization in the nucleus has been associated to an aggressive behavior, our data indicate that in MM cells at resting conditions GSK-3 nuclear functions might not be selected.

GSK-3 was demonstrated to play an important role in modulating cell growth in several solid tumors and two recent studies have shown that B-CLL and Mixed Lineage Leukemia-associated AML cell survival is greatly impaired by the inhibition of GSK-3 [34,49]. Our data indicate that GSK-3 promotes MM cell survival both at basal and under stressed (proteasome inhibition) growing conditions (Fig. 2, 3 and 4). Firstly, the results with the chemical inhibitors SB216763 and SB415286 indicate that GSK-3 inhibition in MM cells leads to a reduction of MM cell growth. This effect is achieved despite the fact that GSK-3 inhibition causes an increase of β-catenin and phospho-ERK levels, which is expected to result in a growth advantage. Moreover, we have observed that exposing MM cells to GSK-3 inhibitors could augment the response to the cytotoxic effects of BZ, a clinically used chief therapeutic agent in MM therapy. BZ regulates several signaling pathways important for cell survival, among which are the AKT, MCL-1, NF-κB and the Wnt/β-catenin-regulated cascades [42]. Our results suggest that GSK-3 might positively regulate the AKT and MCL-1-dependent survival pathways which are normally targeted by BZ, since GSK-3 inhibitors cause a reduction of the BZ-triggered AKT phosphorylation and MCL-1 degradation. However, even if selective and extensively tested, these inhibitors act indistinctly on GSK-3α and GSK-3β, rendering difficult to study isoform-specific functions; additionally, "off-target" GSK-3-independent effects could also occur. Nevertheless, GSK-3α and GSK-3β down modulation by RNA interference in MM cells substantiated the results found with the inhibitors. In fact, our data indicated that GSK-3α and GSK-3β might control MM cell growth, although in a distinct manner. GSK-3β but not GSK-3α knock down caused basal MM cell apoptosis whereas GSK-3α but not GSK-3β knock down was associated with a trend towards increased BZ-induced apoptosis: this would suggest unexpected different roles of the two GSK-3 isoforms in MM cell survival. As mentioned above, the two GSK-3 isoforms can have overlapping functions in regulating the Wnt/β-catenin signaling pathway but not other cascades, such as the NF-κB one, which is mostly dependent on GSK-3β and is critical for MM cell survival [5,16]. Even if the analysis of the pre-transcriptional steps of the NF-κB pathway did not reveal significative differences upon GSK-3α or β inactivation (data not shown), it is still possible, as already reported by other studies [34], that, even in MM cells, GSK-3β regulates NF-κB transcriptional activity directly on DNA. Moreover, our data suggest that GSK-3α might control AKT protein turnover, while GSK-3β could modulate MCL-1 protein stability. Clearly, this latter effect of GSK-3β - already described in previous studies [12] - is in contradiction with the evidence that GSK-3β knock down leads to basal MM cell reduced viability. Other signaling pathways should therefore be involved here. In agreement with previous studies, we have shown that BZ induces a reduction of total AKT (with the paradoxical effect of increasing its Ser 473 phosphorylation) and MCL-1 protein levels in MM cells. Interestingly, these BZ-induced biochemical changes were amplified by the treatment with the GSK-3 inhibitors, which were also able to cause a reduction of AKT Ser 473 phosphorylation (Fig. 4). Remarkably, we demonstrated that the siRNA-mediated knock down of GSK3α and of both the GSK-3 isoforms but not of GSK-3β alone, was associated with the same changes of AKT and MCL-1 levels as seen with the inhibitors. Therefore, our experiments suggest that GSK-3β could influence MM cell survival through MCL-1 and AKT-independent mechanisms, while GSK-3α could modulate BZ-induced MM cell apoptosis interfering with pathways controlling AKT and MCL-1 protein levels. To note, AKT levels have been demonstrated to be controlled by GSK-3 in previous studies [51].

Furthermore, we provided evidence for a functional link between BZ and GSK-3. Indeed, despite the fact that BZ induces Ser 473 AKT phosphorylation, it caused GSK-3 dephosphorylation in Ser 9/21 and its nuclear migration. However, only a little increase in the GSK-3 Tyr phosphorylation (especially in the nucleus) was seen. It is possible that the BZ-induced modifications are not sufficient to trigger a marked Tyr autophosphorylation; alternatively, a full Tyr autophosphorylation could take place at different time points which were not explored in the current study. To note, these alterations of GSK-3 function have also been demonstrated to occur after cell exposure to other apoptotic *noxae *and have been related to the pro-apoptotic effects of this kinase [48]. Interestingly, BZ-induced GSK-3 nuclear accumulation seems to depend on GSK-3 activity, since treatment with SB216763 impaired this intracellular shift. However, the exact significance of GSK-3 nuclear migration upon BZ treatment remains to be elucidated. Since GSK-3 inhibition is accompanied by an increased sensitivity to BZ-triggered cell death, it is unlikely that BZ-induced GSK-3 nuclear accumulation triggers a pro-apoptotic pathway. Oppositely, it is possible that nuclear GSK-3 antagonizes a BZ-dependent nuclear apoptotic pathway. Nonetheless, BZ could trigger GSK-3 activation as a feedback loop to protect MM cells from apoptosis. AKT might be Ser 473 phosphorylated through an unknown, GSK-3-dependent pathway. GSK-3 inhibition would therefore lead to a reduction of phospho-Ser 473 AKT and increased susceptibility to BZ-induced cell death. This unexpected scenario would imply that GSK-3, at basal conditions (to a lesser extent) and in the presence of BZ (to a larger extent), might positively regulate the PI3K/AKT pathway, which in turn is a negative regulator of GSK-3 - as also suggested by the significant amount of GSK-3 (especially GSK-3β) Ser-phosphorylation found in MM cells (Fig. 1A and 6D). In resting conditions this could represent a regulatory loop whereby GSK-3 might buffer down its own activity. However, upon BZ treatment, being Ser 473 AKT phosphorylation increased, other PI3K/AKT-independent mechanism should take place that justify the reduction of GSK-3 Ser-phosphorylation.

## Conclusions

In conclusion, we have herein demonstrated distinct functions of GSK-3α and GSK-3β in MM cell survival. We have employed two strategies to inhibit GSK-3α and β function: the use of selective chemical compounds, which were already extensively characterized, as well as the more specific RNA interference technology. We have shown that GSK3α and β might promote MM cell survival under different conditions and we have described a link between the action of BZ and GSK-3 providing a mechanistic clue to sustain a viewpoint whereby this cross-talk may occur. Our data could be helpful to improve our knowledge of MM pathogenesis and of the mechanisms of response to novel treatments, as well as to develop novel anti-MM targeted therapies.

## Methods

### Patients and cell cultures

Patients were charged to the University of Padova Hospital. Informed consent was obtained from patients according to the declaration of Helsinki and the laboratory protocol was supervised by the institutional scientific review board at the Department of Clinical and Experimental Medicine, University of Padova. Malignant plasma cells were purified using CD138 -coated microbeads (Miltenyi Biotech, Bergish Gladbach, Germany) according to the manufacturer's protocols. Peripheral blood mononuclear cells (PBMC) and bone marrow (BM) mononuclear cells (BMMC) were obtained from PB and BM aspirates of healthy donors and processed as described [52]. Normal plasma cells were generated *in vitro *as previously described [52]. MM cell lines OPM-2 were purchased from the German Collection of Microorganisms and Cell Cultures (DSMZ), RPMI-8226 and U-266 were purchased from the American Type Culture Collection (Rockville, USA); the IL-6-dependent MM cell line INA-6 was a generous gift of Dr. M. Gramatzki, Division of Stem Cell Transplantation and Immunotherapy, University of Kiel, Germany. Cell lines were maintained in RPMI 1640 medium supplemented with 10% fetal bovine serum, L-glutamine, antibiotics (Gibco Laboratories, Grand Island, NY, USA) under controlled-atmosphere in incubators at 37°C in the presence of 5% CO_2_. Quality controls were made every 8 weeks to check for Mycoplasma contamination, ploidy, immunophenotype and cell morphology.

### Chemicals

GSK-3α/β inhibitors SB216763 and SB415286 were purchased from Sigma-Aldrich, Italy. Bortezomib was purchased from LC laboratories, MA, USA.

### GSK-3 activity in cell lysates

One-2 μg of whole cell extracts (WCE) were incubated for 10 min at 30º C with 1 mM GSK-3α/β-specific peptide RRRPASVPPSPSLSRHS(pS)HQRR (Upstate, NY, USA), in the presence of 50 mM Tris-HCl, pH 7.5, 12 mM MgCl_2_, 10 μM [γ-^33^P]ATP (~3000 cpm/pmol) (Amersham Biosciences, UK), 0.1 M NaCl, in a total volume of 20 μl. Samples were spotted onto phospho-cellulose paper and radioactivity was detected by liquid scintillation as previously described [52].

### mRNA silencing

RNA interference was performed by using small interfering RNAs purchased from Dharmacon, USA. Briefly, U-266 cells (2 × 10^6^/ml) were nucleofected with the Amaxa^® ^system according to the manufacturer's instructions with siGLO green scrambled siRNAs, on-Target plus SMART pool oligos against GSK-3α and/or GSK-3β-targeting siRNAs (100 picomoles). (GSK-3α-specific target sequences: CACAAGCUUUAACUGAGA; GAAGGUGACCACAGUCGUA; GAGUUCAAGUUCCCUCAGA; CUGGACCAACUGCAAUAUUG. GSK-3β-specific target sequences: GAUCAUUUGGUGUGGUAUA; GCUAGAUCACUGUAACAUA; GUUCCGAAGUUUAGCCUAU; GCACCAGAGUUGAUCUUUG). Cells were immediately put in pre-warmed RPMI complete medium and left in culture for different time lapses. Cells were then harvested and processed to check GSK-3α and β expression by western blot (WB) analysis.

### Evaluation of growth and apoptosis

In [^3^H]thymidine incorporation assay cells were plated in 96-flat well plates (5 × 10^4^/well) with different concentrations of GSK-3 inhibitors SB216763 or SB415286. After 48 or 72 hours [^3^H]thymidine was added to the cultures (10 μCi/well) for the last 12 hours. The [^3^H]thymidine incorporation was evaluated by scintillation counting by using a top count β-counter (Microbeta Plus; Wallac). For BrdU staining, 2×10^6 ^MM cells were incubated with 10 μM BrdU in PBS for 30 minutes and afterwards ice-cold PBS was added to stop the incorporation. Samples were centrifuged at 409 g for 8 minutes at 4°C and pellets were resuspended and fixed in ethanol 70% in deionized H_2_O for 12 hours. Samples were centrifuged at 409 g for 8 minutes and left in 2 ml of denaturing solution (HCl 2 N) for 10 minutes. Samples were washed, resuspended in Sodium tetraborate 0.1 M, pH 8,5 for 10 minutes, washed and resuspended for 10 minutes in 0.5% BSA and 0.1% Tween 20 in PBS. Cells were then incubated for 45 minutes with an anti-BrdU primary antibody (Sigma Aldrich, Milan, Italy), washed and incubated for 30 minutes with a FITC-conjugated anti-mouse secondary antibody (BD Pharmingen, Italy). After a wash, 200 μl of a solution with 10 mg/ml RNAase and 5 μg/ml di Propidium Iodide was added and samples were analyzed by flow cytometry with FACScalibur and CellQuest^® ^analytic software (Becton Dickinson). Apoptosis was assessed by annexin V/Propidium Iodide staining (BD Pharmingen) or, in separate experiments, by detection of mitochondrial membrane potential [37] using the 5,5',6,6', tetrachloro-1,1',3,3'-tetraethylbenzimidazolyl carbocyanin iodide dye (JC-1) (Trevigen, Germany) according to the manufacturers' instructions. In the described experiments cell death was evaluated by the analysis of Forward/Side scatter fluorescence changes. Fluorescence Activated Cell Sorting (FACS) analysis was performed using a FACS-Calibur Cell Cytometer and the CellQuest^® ^software (Becton-Dickinson, Italy).

### Western blot (WB) and antibodies

Twenty to 40 μg of WCE or nuclear and cytoplasmic fractions were subjected to SDS-PAGE and processed by immuno-blot. Detection was performed using chemiluminescence reaction (Pierce, USA). WCE and nuclear and cytoplasmic fractions were prepared according to standard procedures. Antibodies used: GSK-3α/β, (Santa-Cruz Biotechnology, CA, USA); GSK-3β, β-catenin, phospho-Ser 21/9 GSK-3α/β, PARP, MCL-1, ERK1, 2, phospho-Thr202/Tyr204 ERK1,2, AKT, phospho-Ser 473 AKT (Cell Signaling Technology, MA, USA); Smac/DIABLO (Upstate/Millipore, USA); phospho-Tyr 279/216 GSK3α/β (Abcam, UK); Nucleophosmin (Invitrogen, CA, USA); GAPDH (Ambion, USA); β-actin and α-tubulin (Sigma-Aldrich, Italy);

### Immunofluorescence and confocal microscopy

Preparation of cell samples was done as described [52]. For confocal imaging, a Nikon Eclipse TE300 inverted microscope equipped with a PerkinElmer Ultraview LCI confocal system was employed; excitation was performed using the appropriate laser lines. Magnification was set at 600×, using an oil immersion objective. Antibodies used were: GSK-3α/β, Alexa Fluor^® ^488 goat anti-mouse (Molecular Probes Europe, The Netherlands). To quantify cytosolic and nuclear GSK-3, 200 cells were scored for each different condition and the mean of the percentages of cells with cytosolic only and cytosolic and nuclear GSK-3 was calculated and plotted.

### Statistical analysis

Data obtained were evaluated for their statistical significance with the two-tail paired Student's *t *test or one-way ANOVA and Bonferroni's correction as post-hoc test for experiments with multiple observations. Values were considered statistically significant at *p *values below 0.05.

## Competing interests

The authors declare that they have no competing interests.

## Authors' contributions

FP designed and performed the research, analyzed the data and wrote the paper; SM performed the research, analyzed the data, drafted the paper; CG performed the research, analyzed the data; LQT, BM, LP, A Co, M G, AC performed some experiments of the research. FA, RZ and LT contributed patient samples and clinical inputs. GS supervised research, analyzed the data and edited the paper. All the authors read and approved the final manuscript.

## Pre-publication history

The pre-publication history for this paper can be accessed here:

http://www.biomedcentral.com/1471-2407/10/526/prepub

## References

[B1] Woodgett JR Molecular cloning and expression of glycogen synthase kinase-3/factor A Embo J 1990 9 2431 2438 2164470 10.1002/j.1460-2075.1990.tb07419.x PMC552268

[B2] Patel S Doble B Woodgett JR Glycogen synthase kinase-3 in insulin and Wnt signalling: a double-edged sword? Biochem Soc Trans 2004 32 803 808 10.1042/BST0320803 15494020 PMC4485494

[B3] Clevers H Wnt/beta-catenin signaling in development and disease Cell 2006 127 469 480 10.1016/j.cell.2006.10.018 17081971

[B4] Reya T Clevers H Wnt signalling in stem cells and cancer Nature 2005 434 843 850 10.1038/nature03319 15829953

[B5] Hoeflich KP Luo J Rubie EA Tsao MS Jin O Woodgett JR Requirement for glycogen synthase kinase-3beta in cell survival and NF-kappaB activation Nature 2000 406 86 90 10.1038/35017574 10894547

[B6] Liao X Zhang L Thrasher JB Du J Li B Glycogen synthase kinase-3beta suppression eliminates tumor necrosis factor-related apoptosis-inducing ligand resistance in prostate cancer Mol Cancer Ther 2003 2 1215 1222 14617795

[B7] Song L Zhou T Jope RS Lithium facilitates apoptotic signaling induced by activation of the Fas death domain-containing receptor BMC Neurosci 2004 5 20 10.1186/1471-2202-5-20 15157283 PMC420462

[B8] Ougolkov AV Fernandez-Zapico ME Savoy DN Urrutia RA Billadeau DD Glycogen synthase kinase-3beta participates in nuclear factor kappaB-mediated gene transcription and cell survival in pancreatic cancer cells Cancer Res 2005 65 2076 2081 10.1158/0008-5472.CAN-04-3642 15781615

[B9] Rottmann S Wang Y Nasoff M Deveraux QL Quon KC A TRAIL receptor-dependent synthetic lethal relationship between MYC activation and GSK3beta/FBW7 loss of function Proc Natl Acad Sci USA 2005 102 15195 15200 10.1073/pnas.0505114102 16210249 PMC1257707

[B10] Hongisto V Smeds N Brecht S Herdegen T Courtney MJ Coffey ET Lithium blocks the c-Jun stress response and protects neurons via its action on glycogen synthase kinase 3 Mol Cell Biol 2003 23 6027 6036 10.1128/MCB.23.17.6027-6036.2003 12917327 PMC180950

[B11] Linseman DA Butts BD Precht TA Phelps RA Le SS Laessig TA Bouchard RJ Florez-McClure ML Heidenreich KA Glycogen synthase kinase-3beta phosphorylates Bax and promotes its mitochondrial localization during neuronal apoptosis J Neurosci 2004 24 9993 10002 10.1523/JNEUROSCI.2057-04.2004 15525785 PMC6730230

[B12] Maurer U Charvet C Wagman AS Dejardin E Green DR Glycogen synthase kinase-3 regulates mitochondrial outer membrane permeabilization and apoptosis by destabilization of MCL-1 Mol Cell 2006 21 749 760 10.1016/j.molcel.2006.02.009 16543145

[B13] Shakoori A Ougolkov A Yu ZW Zhang B Modarressi MH Billadeau DD Mai M Takahashi Y Minamoto T Deregulated GSK3beta activity in colorectal cancer: its association with tumor cell survival and proliferation Biochem Biophys Res Commun 2005 334 1365 1373 10.1016/j.bbrc.2005.07.041 16043125

[B14] Ougolkov AV Fernandez-Zapico ME Bilim VN Smyrk TC Chari ST Billadeau DD Aberrant nuclear accumulation of glycogen synthase kinase-3beta in human pancreatic cancer: association with kinase activity and tumor dedifferentiation Clin Cancer Res 2006 12 5074 5081 10.1158/1078-0432.CCR-06-0196 16951223 PMC2692690

[B15] Cao Q Lu X Feng YJ Glycogen synthase kinase-3beta positively regulates the proliferation of human ovarian cancer cells Cell Res 2006 16 671 677 10.1038/sj.cr.7310078 16788573

[B16] Beurel E Jope RS The paradoxical pro- and anti-apoptotic actions of GSK3 in the intrinsic and extrinsic apoptosis signaling pathways Prog Neurobiol 2006 79 173 189 10.1016/j.pneurobio.2006.07.006 16935409 PMC1618798

[B17] Potter M Neoplastic development in plasma cells Immunol Rev 2003 194 177 195 10.1034/j.1600-065X.2003.00061.x 12846815

[B18] Hideshima T Chauhan D Richardson P Mitsiades C Mitsiades N Hayashi T Munshi N Dang L Castro A Palombella V Adams J Anderson KC NF-kappa B as a therapeutic target in multiple myeloma J Biol Chem 2002 277 16639 16647 10.1074/jbc.M200360200 11872748

[B19] Annunziata CM Davis RE Demchenko Y Bellamy W Gabrea A Zhan F Lenz G Hanamura I Wright G Xiao W Dave S Hurt EM Tan B Zhao H Stephens O Santra M Williams DR Dang L Barlogie B Shaughnessy JD Jr Kuehl WM Staudt LM Frequent engagement of the classical and alternative NF-kappaB pathways by diverse genetic abnormalities in multiple myeloma Cancer Cell 2007 12 115 130 10.1016/j.ccr.2007.07.004 17692804 PMC2730509

[B20] Hideshima T Chauhan D Hayashi T Podar K Akiyama M Gupta D Richardson P Munshi N Anderson KC The biological sequelae of stromal cell-derived factor-1alpha in multiple myeloma Mol Cancer Ther 2002 1 539 544 12479272

[B21] Mitsiades CS Mitsiades N Poulaki V Schlossman R Akiyama M Chauhan D Hideshima T Treon SP Munshi NC Richardson PG Anderson KC Activation of NF-kappaB and upregulation of intracellular anti-apoptotic proteins via the IGF-1/Akt signaling in human multiple myeloma cells: therapeutic implications Oncogene 2002 21 5673 5683 10.1038/sj.onc.1205664 12173037

[B22] Qiang YW Kopantzev E Rudikoff S Insulinlike growth factor-I signaling in multiple myeloma: downstream elements, functional correlates, and pathway cross-talk Blood 2002 99 4138 4146 10.1182/blood.V99.11.4138 12010818

[B23] Mitsiades N Mitsiades CS Poulaki V Chauhan D Richardson PG Hideshima T Munshi N Treon SP Anderson KC Biologic sequelae of nuclear factor-kappaB blockade in multiple myeloma: therapeutic applications Blood 2002 99 4079 86 10.1182/blood.V99.11.4079 12010810

[B24] Derksen PW Tjin E Meijer HP Klok MD MacGillavry HD van Oers MH Lokhorst HM Bloem AC Clevers H Nusse R van der Neut R Spaargaren M Pals ST Illegitimate WNT signaling promotes proliferation of multiple myeloma cells Proc Natl Acad Sci USA 2004 101 6122 6127 10.1073/pnas.0305855101 15067127 PMC395933

[B25] Hartmann C A Wnt canon orchestrating osteoblastogenesis Trends Cell Biol 2006 16 151 158 10.1016/j.tcb.2006.01.001 16466918

[B26] Krishnan V Bryant HU Macdougald OA Regulation of bone mass by Wnt signaling J Clin Invest 2006 116 1202 1209 10.1172/JCI28551 16670761 PMC1451219

[B27] Tian E Zhan F Walker R Rasmussen E Ma Y Barlogie B Shaughnessy JD Jr The role of the Wnt-signaling antagonist DKK1 in the development of osteolytic lesions in multiple myeloma N Engl J Med 2003 349 2483 2494 10.1056/NEJMoa030847 14695408

[B28] Gregory CA Gunn WG Reyes E Smolarz AJ Munoz J Spees JL Prockop DJ How wnt signaling affects bone repair by mesenchymal stem cells from the bone marrow Ann N Y Acad Sci 2005 1049 97 106 10.1196/annals.1334.010 15965110

[B29] Gregory CA Green A Lee N Rao A Gunn W The promise of canonical Wnt signaling modulators in enhancing bone repair Drug News Perspect 2006 19 445 452 10.1358/dnp.2006.19.8.1043960 17160144

[B30] Cross DA Alessi DR Cohen P Andjelkovich M Hemmings BA Inhibition of glycogen synthase kinase-3 by insulin mediated by protein kinase B Nature 1995 378 785 789 10.1038/378785a0 8524413

[B31] Zhang HH Lipovsky AI Dibble CC Sahin M Manning BD S6K1 Regulates GSK3 under Conditions of mTOR-Dependent Feedback Inhibition of Akt Mol Cell 2006 24 185 197 10.1016/j.molcel.2006.09.019 17052453 PMC1880887

[B32] Eickholt BJ Walsh FS Doherty P An inactive pool of GSK-3 at the leading edge of growth cones is implicated in Semaphorin 3A signaling J Cell Biol 2002 157 211 217 10.1083/jcb.200201098 11956225 PMC2199247

[B33] Etienne-Manneville S Hall A Cdc42 regulates GSK-3beta and adenomatous polyposis coli to control cell polarity Nature 2003 421 753 756 10.1038/nature01423 12610628

[B34] Ougolkov AV Bone ND Fernandez-Zapico ME Kay NE Billadeau DD Inhibition of glycogen synthase kinase-3 activity leads to epigenetic silencing of nuclear factor kappaB target genes and induction of apoptosis in chronic lymphocytic leukemia B cells Blood 2007 110 735 742 10.1182/blood-2006-12-060947 17463171 PMC1924475

[B35] Smith DG Buffet M Fenwick AE Haigh D Ife RJ Saunders M Slingsby BP Stacey R Ward RW 3-Anilino-4-arylmaleimides: potent and selective inhibitors of glycogen synthase kinase-3 (GSK-3) Bioorg Med Chem Lett 2001 11 635 639 10.1016/S0960-894X(00)00721-6 11266159

[B36] Martin M Rehani K Jope RS Michalek SM Toll-like receptor-mediated cytokine production is differentially regulated by glycogen synthase kinase 3 Nat Immunol 2005 6 777 784 10.1038/ni1221 16007092 PMC1933525

[B37] Cossarizza A Salvioli S Flow cytometric analysis of mitochondrial membrane potential using JC-1 Curr Protoc Cytom 2001 Chapter 9 Unit 9 14 18770751 10.1002/0471142956.cy0914s13

[B38] Cole A Frame S Cohen P Further evidence that the tyrosine phosphorylation of glycogen synthase kinase-3 (GSK3) in mammalian cells is an autophosphorylation event Biochem J 2004 377 249 255 10.1042/BJ20031259 14570592 PMC1223856

[B39] Lochhead PA Kinstrie R Sibbet G Rawjee T Morrice N Cleghon V A chaperone-dependent GSK3beta transitional intermediate mediates activation-loop autophosphorylation Mol Cell 2006 24 627 633 10.1016/j.molcel.2006.10.009 17188038

[B40] Zhai P Gao S Holle E Yu X Yatani A Wagner T Sadoshima J Glycogen synthase kinase-3alpha reduces cardiac growth and pressure overload-induced cardiac hypertrophy by inhibition of extracellular signal-regulated kinases J Biol Chem 2007 282 33181 33191 10.1074/jbc.M705133200 17855351

[B41] Rehani K Wang H Garcia CA Kinane DF Martin M Toll-like receptor-mediated production of IL-1Ra is negatively regulated by GSK3 via the MAPK ERK1/2 J Immunol 2009 182 547 553 19109187 10.4049/jimmunol.182.1.547 PMC2850057

[B42] Ocio EM Mateos MV Maiso P Pandiella A San-Miguel JF New drugs in multiple myeloma: mechanisms of action and phase I/II clinical findings Lancet Oncol 2008 9 1157 1165 10.1016/S1470-2045(08)70304-8 19038762

[B43] Hideshima T Mitsiades C Tonon G Richardson PG Anderson KC Understanding multiple myeloma pathogenesis in the bone marrow to identify new therapeutic targets Nat Rev Cancer 2007 7 585 598 10.1038/nrc2189 17646864

[B44] Hideshima T Catley L Raje N Chauhan D Podar K Mitsiades C Tai YT Vallet S Kiziltepe T Ocio E Ikeda H Okawa Y Hideshima H Munshi NC Yasui H Richardson PG Anderson KC Inhibition of Akt induces significant downregulation of survivin and cytotoxicity in human multiple myeloma cells Br J Haematol 2007 138 783 791 10.1111/j.1365-2141.2007.06714.x 17760810

[B45] Podar K Gouill SL Zhang J Opferman JT Zorn E Tai YT Hideshima T Amiot M Chauhan D Harousseau JL Anderson KC A pivotal role for Mcl-1 in Bortezomib-induced apoptosis Oncogene 2008 27 721 731 10.1038/sj.onc.1210679 17653083

[B46] Chen KF Yeh PY Yeh KH Lu YS Huang SY Cheng AL Down-regulation of phospho-Akt is a major molecular determinant of bortezomib-induced apoptosis in hepatocellular carcinoma cells Cancer Res 2008 68 6698 6707 10.1158/0008-5472.CAN-08-0257 18701494

[B47] Chen KF Yeh PY Hsu C Hsu CH Lu YS Hsieh HP Chen PJ Cheng AL Bortezomib overcomes tumor necrosis factor-related apoptosis-inducing ligand resistance in hepatocellular carcinoma cells in part through the inhibition of the phosphatidylinositol 3-kinase/Akt pathway J Biol Chem 2009 284 11121 11133 10.1074/jbc.M806268200 19261616 PMC2670117

[B48] Bijur GN Jope RS Proapoptotic stimuli induce nuclear accumulation of glycogen synthase kinase-3 beta J Biol Chem 2001 276 37436 37442 10.1074/jbc.M105725200 11495916 PMC1973163

[B49] Wang Z Smith KS Murphy M Piloto O Somervaille TC Cleary ML Glycogen synthase kinase 3 in MLL leukaemia maintenance and targeted therapy Nature 2008 455 1205 1209 10.1038/nature07284 18806775 PMC4084721

[B50] Force T Woodgett JR Unique and overlapping functions of GSK-3 isoforms in cell differentiation and proliferation and cardiovascular development J Biol Chem 2009 284 9643 9647 10.1074/jbc.R800077200 19064989 PMC2665084

[B51] Nemoto T Kanai T Yanagita T Satoh S Maruta T Yoshikawa N Kobayashi H Wada A Regulation of Akt mRNA and protein levels by glycogen synthase kinase-3beta in adrenal chromaffin cells: effects of LiCl and SB216763 Eur J Pharmacol 2008 586 82 89 10.1016/j.ejphar.2008.02.075 18395711

[B52] Piazza FA Ruzzene M Gurrieri C Montini B Bonanni L Chioetto G Di Maira G Barbon F Cabrelle A Zambello R Adami F Trentin L Pinna LA Semenzato G Multiple myeloma cell survival relies on high activity of protein kinase CK2 Blood 2006 108 1698 1707 10.1182/blood-2005-11-013672 16684960

